# Sertraline-induced fetotoxicity in mice: Protective effects of vitamin E on foetal growth and organ histology

**DOI:** 10.6026/973206300223060

**Published:** 2026-05-31

**Authors:** Amit Kumar Nayak, Deepa Devadas, Atul Kumar Singh, Chetan Sahni, Anand Mishra, Jawed Akhtar Md

**Affiliations:** 1 Department of Anatomy, Institute of Medical Sciences, Banaras Hindu University, Varanasi, Uttar Pradesh, India; 2 Department of Anaesthesiology, Institute of Medical Sciences, Banaras Hindu University, Varanasi, Uttar Pradesh, India; 3 Department of Anatomy, All India Institute of Medical Sciences, Gorakhpur, Uttar Pradesh, India; 4 Department of Anatomy, Indira Gandhi Institute of Medical Sciences, Patna, Bihar, India

**Keywords:** Sertraline, Fetotoxicity, pregnancy, vitamin E, oxidative stress, foetal development, swiss albino mice, histopathology

## Abstract

Sertraline, a selective serotonin reuptake inhibitor (SSRI), is widely used for treating depression and anxiety disorders, including during pregnancy, yet its potential effects on fetal development remain a concern. Oxidative stress is considered a possible mechanism for drug-induced developmental toxicity, suggesting antioxidant supplementation may mitigate such effects. Therefore, it is of interest to assess the fetotoxic potential of sertraline and the protective role of Vitamin E in eighteen pregnant Swiss albino mice divided into control, sertraline-treated (60 mg/kg/day) and sertraline + Vitamin E (100 mg/kg/day) groups. Treatments were given orally from gestational day 6-15, followed by evaluation of fetal growth and organ histopathology on day 19. Sertraline exposure significantly reduced fetal number, weight, crown-rump length and placental metrics, with marked cardiac, hepatic and renal alterations, while Vitamin E co- administration partly ameliorated these effects. Thus, we show oxidative stress in sertraline-induced fetal toxicity and demonstrating the partial protective potential of Vitamin E supplementation.

## Background:

Mental health disorders such as anxiety and depression represent a growing global health concern. The prevalence of these conditions has increased substantially in recent decades due to factors such as chronic stress, lifestyle changes, sleeps disturbances and increased social media addiction [1]. Excessive use of social media platforms has been associated with increased level of anxiety and depression, along with reduced academic self-efficacy, weaker social belongingness and lower overall life satisfaction. Among different population groups, women of reproductive age are particularly affected and many require pharmacological treatment during pregnancy [2]. Sertraline is one of the most commonly prescribed selective serotonin reuptake inhibitors (SSRIs) for the treatment of depression and anxiety disorders [3]. Most of the preclinical studies have stated sertraline to be a safe drug, while some recent studies have raised concern over its use during pregnancy [4]. Therefore, it is of interest to evaluate the teratogenic effects of Sertraline in mice and to investigate the possible protective role of Vitamin E co-administration in ameliorating these effects.

## Materials and Methods:

The present study was conducted in the Department of Anatomy, Institute of Medical Sciences, Banaras Hindu University, Varanasi and Uttar Pradesh, India.

## Procurement and housing of animals:

Eighteen female Swiss albino mice, weighing approximately around 18-20 g and aged 8-12 weeks, were acquired from the departmental animal house after receiving approval from the Institutional Animal Ethics Committee (IAEC), IMS, BHU (letter No. Dean/2023/IAEC/6187). The mice were housed in pairs in polypropylene cages with husk bedding. Each mouse was uniquely marked with picric acid on different parts for identification. The cages were maintained in a controlled environment within the departmental animal house. The room temperature was maintained at 25°C ± 1 °C. A relative humidity of 55 ± 5% was maintained using an in-house humidifier. A 12 hr: 12 hr light-dark cycle was maintained throughout the study. Mice were provided with pelleted diet twice daily. A glass bottle containing tap water was fixed in a provided slot in mouse cage to provide unrestricted access to tap water. For mating, female mice were paired with the male mice in a ratio of 2 females to 1 male during evening hours. The following morning, the females were examined for the presence of a vaginal plug. The presence of the vaginal plug was further confirmed by preparing a vaginal smear and examining it under a microscope. After confirmation of mating, the pregnant females were transferred to separate cages. The cages were properly marked and the day of confirmation was considered as gestational day zero (GD 0). The body weight of the pregnant mice was measured daily using a digital weighing balance.

## Procurement of drug and dose calculation: 

Sertraline was commercially obtained under the brand name Carelin-50 (Ulteum Pharma Pvt. Ltd). Each tablet contained 50 mg of sertraline. The maximum human dose permitted by FDA is 200 mg/day, which amounts to 3.33 mg/kg body weight/ day. The calculated animal equivalent dose calculated was 42 mg/kg body weight/ day (calculation explained below) [5]. In the present study, the administered dose given was 1.5 times the maximum therapeutic dose, which was calculated to be 60 mg/kg body weight/ day in mice. Sertraline was administered orally using a gavage syringe from day six till day fifteen [5].

## Dose calculation:

Therapeutic dose range of sertraline in human is 50-200 mg/day.

The approved Maximal dose is 200 mg/day,

Which converts to 3.33mg/kg body weight /day?

Now,

Animal equivalent dose (AED)= Human equivalent dose (HED)x(Human Km)/(Animal Km)

Where, Km is species constant.

(Human Km)/(Mice Km) = 12.3

So,

Mouse equivalent dose (AED)=3.33 x12.3 mg/ kg body weight / day

=40.96 ≈41 mg/ kg body weight / day

Dose to be given is 1.5 times this dose, which amounts to,

41 x1.5 ≈60 mg/ kg body weight / Day

Each mouse was weighed before the start of sertraline administration and was reweighed daily before dosing to ascertain optimal dosing as per protocol. Vitamin E was commercially obtained as Vitamin E Capsule USP 400 mg, manufactured by Merck Ltd (CM Care) under trade name of Evion 400. As it is a fat-soluble substance, it was given at a dose of 100 mg/kg body weight/ day via gavage after mixing with soyabean oil, using a separate round-tipped mouse gavage needle [6, 7]. Vitamin E was administered one hour after the administration of sertraline. Soyabean oil, used as vehicle for Vitamin E administration, was obtained commercially under the brand name of "Fortune soya health", manufactured by Adani Wilmar Ltd (Gujrat, India).

## Experimental groups:

Eighteen mice were divided into three groups; each group was assigned six mice.

[1] Group-1: Control group of pregnant dams (given equivalent amount of saline and soyabean oil from day six till day fifteen of pregnancy).

[2] Group- 2: Pregnant mice given sertraline (60 mg/ kg body weight/ day) and soyabean oil orally from day six till day fifteen of pregnancy).

[3] Group-3: Pregnant mice receiving sertraline (60 mg/kg body weight/ day) and Vitamin E (100 mg/ kg body weight/ day) from day six till day fifteen of pregnancy.

On 19th day of gestation (GD 19), the pregnant mice were sacrificed under deep ether anaesthesia. After sacrifice, a midline abdominal incision was made. Both uterine horns were exposed and carefully examined for any visible abnormalities. An uterotomy was then performed to remove the foetuses. The gestational sacs were thoroughly examined using a magnifying lens to identify any signs of resorption. The viable foetuses were placed on a tissue paper and gently bloated dry. Each foetus was carefully examined for external abnormalities like haemorrhage, cleft lip, cleft palate, limb deformities, or other craniofacial anomalies. Photograph of any visible defects were taken immediately. Dried foetuses were then placed on a graph paper and the points corresponding to the vertex of the head and the tip of coccyx were marked. The crown-rump length (CRL) was calculated by counting the squares between these two marked points. The weight of each foetus was measured using a digital weighing machine with accuracy up to 0.001gms. After gross examination, photography, measuring CRL and weighing, the foetuses were preserved in 10% neutral buffered formalin solution for fixation. Following fixation, the liver, heart and kidneys of the foetuses were dissected. These organs were then used for further histological examination.

## Statistical analysis:

Statistical analysis was performed using one-way analysis of variance (ANOVA) followed by Tukey's post-hoc test for multiple group comparisons. Differences between groups were considered statistically significant when the p value was less than 0.05. Statistical analysis was performed using SPSS software (version 20).

## Histological photography:

The photomicrography for histological observation was taken with the help of digital microscope fitted with USB camera (MOTIC 2000, 1:3).

## Results:

**Figure 1:**
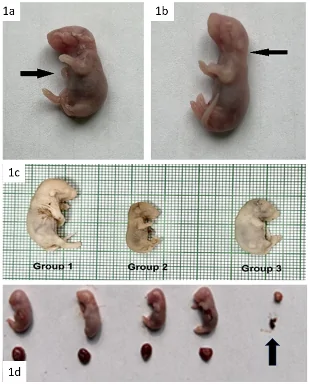
Gross foetal findings, 1a) Flexion deformity in limb in fetus of Group-2; 1b) Extension deformity in neck Group-2; 1c) Comparison of crown-rump length across all groups; 1d) Fetal resorption in Group-2

The total number of fetuses born in Group 1, Group 2 and Group 3 were 67, 54 and 64, respectively. The control group (Group 1) showed the highest average number of fetuses per litter, calculated as 11.17 ± 0.98. In Group 3, the average number of fetuses was slightly lower at 10.67 ± 1.03. The lowest average fetuses were observed in sertraline treated group, with of 9 ± 1.1 fetuses. In contrast, the trend was reversed when considering the average number of resorptions. The mean number of resorptions was 0.17 ± 0.41 in Group 1, 1 ± 0.89 in Group 2 and 0.33 ± 0.52 in Group 3, respectively. A statistically significant decrease in number of fetuses was observed in Group-2 compared to Group-1 (p < 0.01) (Table 1). No gross abnormalities such as cleft lip, cleft palate or other craniofacial deformities were observed in any of the groups (Figure 1). However, flexion deformities of the limbs and extension deformities of the neck were observed in few fetuses. The average fetal weight in Group 1 was 1.362 ± 0.953g, which was the highest among the three groups. In comparison, the mean fetal weight in Group 2 was 0.885 ± 0.131 g, while in Group 3 it was 1.243 ± 0.36 g. A statistically highly significant reduction in fetal weight was observed in Group 2 compared with Group 1, as well as between Group 2 and Group 3 (p<0.01). Additionally, a significant difference was noted between Group 1 and Group 3 (p<0.05) (Table 1). The mean fetal crown-rump length (CRL) was 2.223 ± 0.162 cm in Group 1, 1.605 ± 0.171 cm in Group 2 and 2.143 ± 0.145 cm in Group 3. The CRL was lowest in the sertraline-treated (Group 2). Statistical analysis revealed a significant difference in fetal CRL among the three groups (p<0.05) (Table 1). The mean placental diameter was 0.141 ± 0.008 in Group 1, 0.0841 ± 0.015 in Group 2 and 0.0814 ± 0.008g in Group 3. The average placental diameter was highest in Group 1 (8.507 ± 0.659mm) and lowest in Group 2 (6.667 ± 0.932 mm). In Group 3, the mean placental diameter was 7 ± 0.816 mm. One-way ANOVA showed that the difference in placental weight between Group 1 and Group 2, as well as between Group 1 and Group 3, was highly significant (p < 0.01). However, the difference between Group-2 and Group-3 was not statistically significant. Similar findings were observed for placental diameter. The difference was statistically significant between Group 1 and Group 2 and between Group 1 and Group 3 (p < 0.01), whereas the difference between Group 2 and Group 3 was not significant (Table 2).

**Figure 2:**
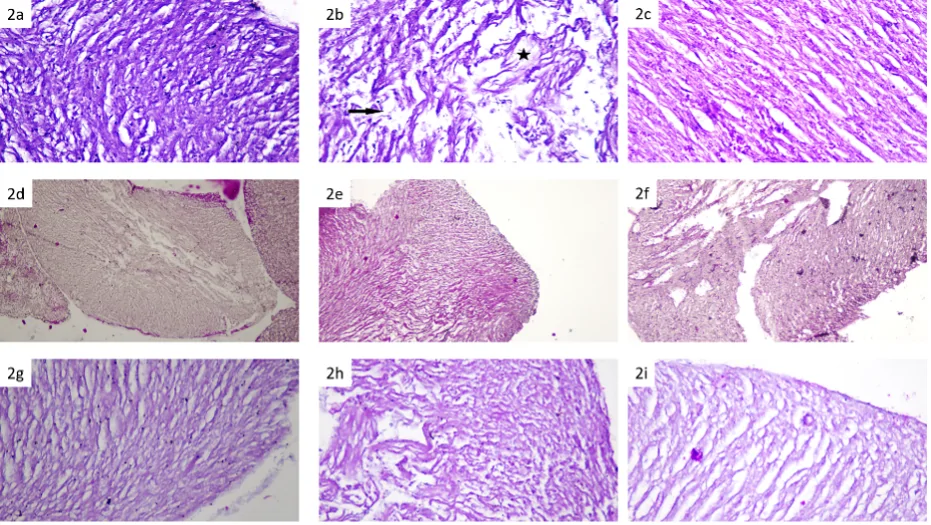
Histological features of foetal heart across groups. 2a) Group-1 fetal heart showing normal myocardial branching pattern and normal cellular architecture (H&E, 400X); 2b) Group-2 fetal heart showing loss of branching pattern (→) and cellular debris (*) (H&E, 400X); 2c. Group-3 fetal heart showing normal myocardial density and branching (H&E, 400X); 2d) Group-1 fetal heart showing no obvious fibrosis (MT, 100X); 2e & 2f) Group-2 & Group-3 fetal heart showing no obvious fibrosis (MT, 100X); 2g: Group-1 fetal heart (PAS, 400X); 2h & 2i) Group-2 & Group-3 fetal heart exhibiting normal polysaccharide content as compared to control fetal heart (PAS, 400X)

**Figure 3:**
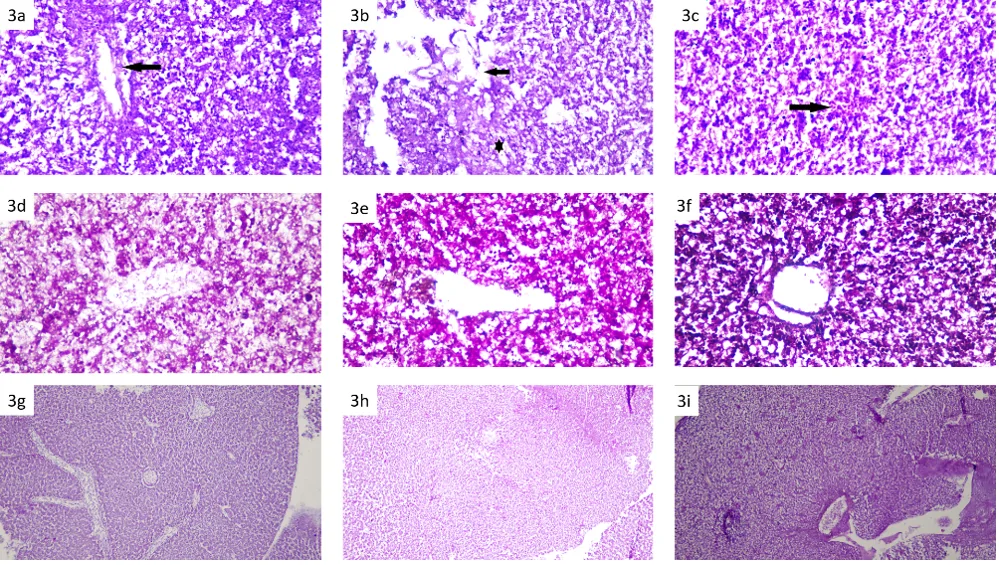
Histological features of foetal liver across groups. 3a) Group-1 (Control) fetal liver showing normal cellular architecture and a developing central vein (left arrow) (H&E, 400X); 3b) Group-2 fetal liver showing empty lacunar space (left arrow) and cell necrosis (*) (H&E, 400X); 3c) Group-3 fetal liver showing aggregation of red blood cells to form primitive sinusoids (→) (H&E, 400X); 3d. Group-1 (Control) fetal liver showing no abnormal fibrosis (MT, 400X); 3e & 3f: Group-2 & Group-3 fetal liver showing no abnormal fibrosis around central vein (MT, 400X); 3g: Group-1 (Control) fetal liver (PAS, 100X); 3h & 3i) Group-2 & Group-3 fetal liver having similar staining comparable to control fetal liver (PAS, 100X).

**Figure 4:**
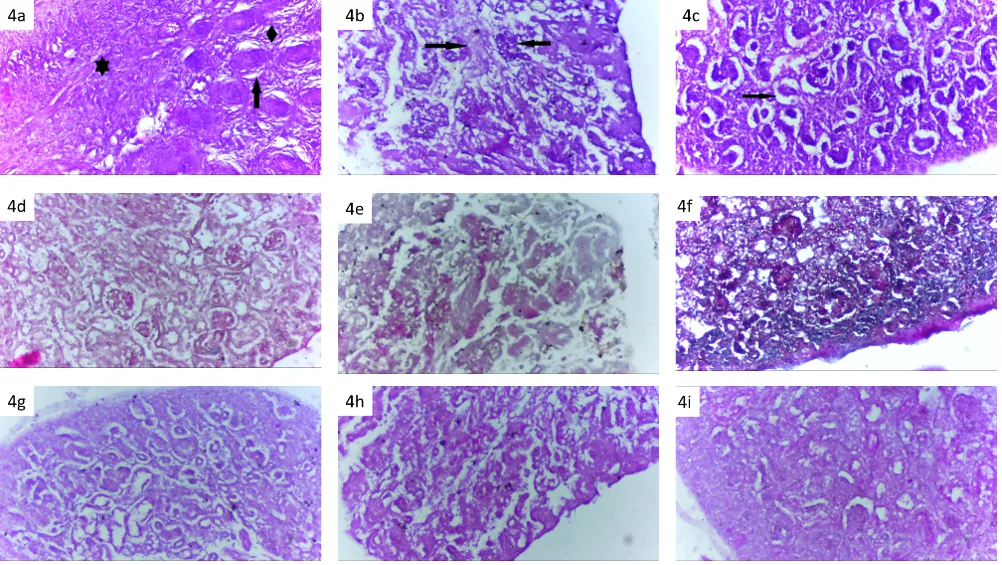
Histological features of foetal kidney across groups. 4a) Group-1 fetal kidney showing demarcation into outer cortex (diamond) and inner medulla (*). Forming glomerulus with glomerular space can be seen (up arrow). (H&E, 100X); 4b) Group-2 fetal kidney showing loss of division into cortex at medulla. Necrosis (→) and clumping of cells (left arrow) can be seen (H&E, 100X); 4c) Group-3 fetal kidney showing developing glomerulus surrounded by per glomerular space can be seen (H&E, 100X); 4d) Group-1 fetal control kidney showing no pathological fibrosis. (MT, 100X); 4e) Group-2 fetal kidney showing some fibrosis. (MT, 100X); 4f) Group-3 fetal kidney showing interstitial fibrosis. (MT, 100X); 4g) Group-1 fetal control kidney. (PAS, 100X); 4h & 4i) Group-2 & Group-3 fetal kidney showing similar PAS staining comparable to control. (PAS, 100X).

When compared to normal fetal heart (Figure 2), in Group 2 (Sertraline-treated), a reduction in myocardial abundance along with abnormal branching patterns was observed (Figure 2). At higher magnification with hematoxylin and eosin (H & E) staining, areas suggestive of cell death with accumulation of cellular debris were noted. Clusters of inflammatory cells were also observed in several regions. However, MT staining did not demonstrate any evidence of fibrosis and the PAS staining intensity was comparable to that observed in the control group (Figure 2). The fetal hearts in Group 3 (Sertraline + Vitamin E) showed architectural features comparable to those observed in the control group. The myocardial arrangement and branching patterns appeared normal. MT and PAS staining did not reveal any abnormalities (Figure 2). When compared to normal fetal liver (Figure 3), Group 2 (Sertraline-treated) exhibited several pathological changes in the fetal liver (Figure 3). Areas of cellular necrosis resulting in empty lacunar spaces, inflammatory cell infiltration, rupture of blood vessels, hemorrhage and loss of normal hepatic architecture were observed at multiple sites, indicating severe hepatic toxicity. However, no pathological fibrosis or difference in staining intensity could be appreciated in MT or PAS staining (Figure 3). In Group 3 (Sertraline + Vitamin E) normal architecture of developing liver was maintained. However, focal areas of interstitial hemorrhage were observed at some sites. MT and PAS staining patterns were also similar to those in the control group (Figure 3). When compared to normal fetal kidney (Figure 4), Group 2 (Sertraline-treated) exhibited marked histopathological alterations in the fetal kidneys (Figure 4). There was a complete loss of differentiation between the cortex and medulla, along with areas of necrosis and clumping of cells in H & E staining. Numerous empty lacunar spaces were also seen, likely resulting from cellular necrosis. Mild fibrosis was observed in MT-stained slide. However, PAS staining showed affinity pattern comparable to that observed in the control kidney (Figure 4). In Group 3 (Sertraline + Vitamin E) no major abnormalities were observed on H & E and PAS staining. However, MT staining showed increased interstitial fibrosis in certain areas (Figure 4).

## Discussion:

The world is witnessing a continuous rise in the number of individuals suffering from anxiety, depression and other related mental disorders [8]. The use of therapeutic intervention is also increasing with it. Sertraline, one of the most widely prescribed drugs in this context, belongs to the class of antidepressant and anxiolytic medications known as selective serotonin reuptake inhibitors (SSRIs) [3]. It is most widely prescribed SSRIs for elderly patients and for the management of perinatal depression [9, 10]. In addition to treating depression and anxiety disorders, sertraline is also used in the management of a broad range of psychiatric conditions [11]. Sertraline is typically prescribed in doses of 50 to 200 mg per day in adults and this dosage range is generally considered clinically safe and well tolerated [12]. The present study explored the effects of a higher dose of sertraline (60 mg/kg body weight/ day) on fetuses of Swiss albino mice. A statistically significant reduction in the number of fetuses was observed in the sertraline-treated group compared with the control group, suggesting an increase rate of fetal loss. An increase in fetal resorption was also observed in the sertraline-treated group, although this difference was not statistically significant. Additionally, fetal weight and crown-rump length (CRL) were found to be significantly reduced in the sertraline- treated group. A significant reduction in placental weight and placental diameter was observed in the sertraline-treated group. However, these parameters showed significant improvement when Vitamin E was co-administered, indicating a possible protective effect. Overall, the findings of this study suggests that sertraline administered at a dose of 60 mg/ kg body weight/ day produces phytotoxic effects and that co-administration of Vitamin E may ameliorate these toxic effects in pregnant dams. Several studies have reported findings similar to those observed in the present study such as, significant decrease in fetal weight and an increased resorption rate following administration of sertraline at a dose of 60 mg/kg body weight/ day in mice. There is documentation of several gross congenital abnormalities, including cleft palate, exophthalmia, renal deformities, ventricular hemorrhage and body wall hemorrhage as well.

The authors have attributed the phytotoxic effects not on the dose of sertraline but the property of sertraline to increase the concentration of serotonin. It has been opined that serotonin by controlling gene expression acts a regulator for cell proliferation, migration and differentiation [4, 13]. Histopathological examination provided additional evidence of sertraline-induced toxicity. Heart of fetuses born of dams treated with sertraline-only during pregnancy exhibited various abnormalities on histological examinations. There is a decrease in density and branching pattern of myocardial cells. There is evidence of myocardial cell necrosis. These structural changes in heart were absent in Group 3 fetuses, where dams were administered Vitamin E along with sertraline. A similar study in mice reported decreased myocardial proliferation and functioning in neonatal hearts associated with perinatal sertraline exposure. Increased cardiac fibrosis on MT staining was also mentioned. The researchers hypothecated sertraline induced decreased in 5-HT2B receptor mRNA to be the pivotal mechanism behind cardiotoxicity [14]. Exposure to sertraline in prenatal period leads to defective cardiomyocyte growth particularly in left ventricle producing - left heart syndrome [15]. Serotonin and its binding protein act as a major morph genic signal in cardiac development, particularly in development of endocardial cushion [16]. A study on rats has reported that there was significant rise in various cardiac inflammatory markers (AST, CK-MB, cTn-T and LDH). Authors opined that generation of oxygen free radical due to sertraline administration is the mechanism behind the cardiotoxicity of the drug [17]. Another study found that sertraline administration and consequent elevation in reactive oxygen species led to suppression of critical calcium regulating genes and up regulation of genes of neonatal cardiac tissue that take part in serotonin signaling leading to cardiac malformations [18]. In fetal liver, sertraline exposure resulted in hepatocellular necrosis, loss of architecture and infiltration of inflammatory cells. These changes were reversed to many extents in Group 3 mice. Similar hepatic findings were observed by various authors and they have attributed it to free radical induced damages to hepatic parenchyma and to increase in level of nitrous oxide (NO) [19, 20]. Absence of such findings when treated with Vitamin E, a potent antioxidant further strengthened the free radical induced damage hypothesis. Fetal kidney of Group 2 depicted a complete loss of differentiation into cortex at medulla along with necrosis and clumping of cells in H & E staining. In MT staining, mild fibrosis was observed, finding very similar to previous studies [20]. Authors have opined oxygen free radical and formation of dynamin complex in Bowman's capsule was also opined as one of the causes for nephrotoxicity [21]. Taken together, the findings of this study suggest that exposure to high doses of sertraline during pregnancy may adversely affect fetal development, possibly through oxidative stress-mediated mechanisms. The protective role of Vitamin E supplementation suggests that antioxidant therapy is useful in mitigation of sertraline induced toxicity.

## Conclusion:

High-dose sertraline exposure during pregnancy produced fetotoxic effects in mice, evidenced by impaired fetal growth, altered placental morphology and histopathological damage to the developing heart, liver and kidneys. Co-administration of Vitamin E significantly ameliorated these adverse outcomes, suggesting its antioxidant properties may help counteract sertraline-induced oxidative stress. Thus, we show the role of oxidative mechanisms in antidepressant-related fetal toxicity and highlight the need for further studies to clarify underlying pathways and explore safe protective interventions.

## Funding:

This research received no external funding.

## Figures and Tables

**Table 1:** Number of dams, number of fetuses, number of resorption, number of fetuses, mean fetal weight, means fetal crown-rump length, mean placental weight and Mean Placental diameter in Group 1, Group 2 and Group 3

**Group**	**Number Of Dams**	**Number of fetuses (Mean ± SD)**	**Number of resorptions (Mean ± SD)**	**Number of Fetus (N)**	**Mean Fetal weight (gm)**	**Mean Fetal Crown-rump length (cm)**	**Mean Placental weight (gm)**	**Mean Placental diameter (mm)**
Group 1	6	11.17 ± 0.98	0.17 ± 0.41	67	1.362 ± 0.095	2.223 ± 0.162	0.141 ± 0.008	8.507 ± 0.659
Group 2	6	9 ± 1.1	1 ± 0.89	54	0.885 ± 0.131	1.605 ± 0.171	0.0841 ± 0.015	6.667 ± 0.932
Group 3	6	10.67 ± 1.03	0.33 ± 0.52	64	1.243 ± 0.36	2.143 ± 0.145	0.0814 ± 0.008	7 ± 0.816

**Table 2:** One way ANOVA (Post Hock, Tukey) test for comparison of number of foetuses, resorptions, Placental diameter, placental weight, crown-rump length and weight of the foetuses in Group 1, Group 2 and Group 3 (* Significant at p < 0.05, ** Significant at p < 0.01)

**Groups/Variable**	**Number of fetuses (significance)**	**Number of resorptions (significance)**	**Placental diameter (significance)**	**Placental Weight (significance)**	**Crown-rump length of the fetuses (significance)**	**Weight of the fetuses (significance)**
Group 1 vs Group 2	0.007**	0.095	0.000**	0.000**	0.000**	0.000**
Group 2 vs Group 3	0.035*	0.203	0.065	0.345	0.000**	0.000**
Group 1 vs Group 3	0.688	0.895	0.000**	0.000**	0.012*	0.01*
